# Gene Expression Profiling on Global cDNA Arrays Gives Hints Concerning Potential Signal Transduction Pathways Involved in Cardiac Fibrosis of Renal Failure

**DOI:** 10.1002/cfg.347

**Published:** 2003-12

**Authors:** Kerstin Amann, Heidrun Ridinger, Christiane Rutenberg, Eberhard Ritz, Gerhard Mall, Christian Maercker

**Affiliations:** 1 Department of Pathology University of Erlangen-Nürnberg Krankenhausstrasse 8-10 Erlangen D-91054 Germany; 2 Resource Center for Genome Research Im Neuenheimer Feld 280 Heidelberg D-69120 Germany; 3 Department of Internal Medicine University of Heidelberg Bergheimerstrasse 56a Heidelberg 69115 Germany; 4 Department of Pathology Grafenstrasse 9 Darmstadt 64283 Germany

## Abstract

Cardiac remodelling with interstitial fibrosis in renal failure, which so far is only poorly understood on the molecular level, was investigated in the rat model by a global gene expression profiling analysis. Sprague–Dawley rats were subjected to subtotal nephrectomy (SNX) or sham operation (sham) and followed for 2 and 12 weeks, respectively. Heart-specific gene expression profiling, with RZPD Rat Unigene-1 cDNA arrays containing about 27 000 gene and EST sequences revealed substantial changes in gene expression in SNX compared to sham animals. Motor protein genes, growth and differentiation markers, and extracellular matrix genes were upregulated in SNX rats. Obviously, not only genes involved in cardiomyocyte hypertrophy, but also genes involved in the expansion of non-vascular interstitial tissue are activated very early in animals with renal failure. Together with earlier findings in the SNX model, the present data suggest the hypothesis that the local renin–angiotensin system (RAS) may be activated by at least two pathways: (a) via second messengers and Gproteins (short-term signalling); and (b) via motor proteins, actins and integrins (longterm signalling). The study documents that complex hybridization analysis yields reproducible and promising results of patterns of gene activation pointing to signalling pathways involved in cardiac remodelling in renal failure. The complete array data are available via http://www.rzpd.de/cgi-bin/services/exp/viewExpressionData.pl.cgi


**Comparative and Functional Genomics**
*Comp Funct Genom* 2003; **4**: 571–583.
Published online in Wiley InterScience (www.interscience.wiley.com). **DOI:** 10.1002/cfg.347

**Primary Research Paper**

### Gene expression profiling on global cDNA arrays gives hints concerning potential signal transduction pathways involved in cardiac fibrosis of renal failure

Kerstin Amann[1]*, Heidrun Ridinger[2], Christiane Rutenberg[2], Eberhard Ritz[3], Gerhard Mall[4] and Christian Maercker[2]

[1] *Department of Pathology, University of Erlangen, Krankenhausstr. 8-10, D-91054 Erlangen, Germany*
[2] *Resource Center for Genome Research, Im Neuenheimer Feld 280, D-69120 Heidelberg, Germany*
[3] *Department of Internal Medicine, University of Heidelberg, Bergheimerstrasse 56a, 69115 Heidelberg, Germany*
[4] *Department of Pathology, Grafenstrasse 9, 64283 Darmstadt, Germany*

**Correspondence to:*
*Kerstin Amann, Department of Pathology, University of Erlangen-Nürnberg, Krankenhausstrasse 8-10, D-91054 Erlangen, Germany.*
*E-mail: kerstin.amann@patho.imed.uni-erlangen.de*

### Abstract

**Cardiac remodelling with interstitial fibrosis in renal failure, which so far is only poorly understood on the molecular level, was investigated in the rat model by a global gene expression profiling analysis. Sprague–Dawley rats were subjected to subtotal nephrectomy (SNX) or sham operation (sham) and followed for 2 and 12 weeks, respectively. Heart-specific gene expression profiling, with RZPD Rat Unigene-1 cDNA arrays containing about 27 000 gene and EST sequences revealed substantial changes in gene expression in SNX compared to sham animals. Motor protein genes, growth and differentiation markers, and extracellular matrix genes were upregulated in SNX rats. Obviously, not only genes involved in cardiomyocyte hypertrophy, but also genes involved in the expansion of non-vascular interstitial tissue are activated very early in animals with renal failure. Together with earlier findings in the SNX model, the present data suggest the hypothesis that the local renin–angiotensin system (RAS) may be activated by at least two pathways: (a) via second messengers and G-proteins (short-term signalling); and (b) via motor proteins, actins and integrins (long-term signalling). The study documents that complex hybridization analysis yields reproducible and promising results of patterns of gene activation pointing to signalling pathways involved in cardiac remodelling in renal failure. The complete array data are available via http://www.rzpd.de/cgi-bin/services/exp/viewExpressionData.pl.cgi**
**Copyright © 2003 John Wiley & Sons, Ltd.**

Received: 23 April 2003
Revised: 3 September 2003
Accepted: 10 October 2003

**Keywords:** **left ventricular hypertrophy; cardiac fibrosis; renal failure; gene expression profiling; renin–angiotensin system; endothelin**

### Introduction

Death from cardiac causes is up to 20 times more frequent in patients with renal failure than in the background population (Raine *et al.*, 1992; US Renal Data System, 1995). Patients with renal failure are the group with the highest known cardiovascular risk. There is increasing evidence that such a risk is not unique to endstage renal failure, but may occur very early on in the course of renal disease (Sharma *et al.*, 1996). The cardiovascular lesions include complex cardiac remodelling affecting not only contractile parenchyma with development of left ventricular hypertrophy (LVH), but also vascular and interstitial tissue (Amann and Ritz, 1997, 2001).

Cardiac remodelling in renal failure is of interest not only because of its clinical relevance, but

Copyright © 2003 John Wiley & Sons, Ltd.

also because it may provide experimental advantages, since the onset of the abnormality is clearly defined in time and primary manipulations of the heart are not required. Thus, renal failure may be a useful model for the more general problem of identifying mechanisms underlying cardiac remodelling. The exact nature of these cardiac-specific structural alterations have been characterized in detail in patients with renal failure (Amann *et al.*, 1998) and particularly in the animal model of the subtotally nephrectomized rat (SNX), which is a well-accepted model of moderate renal failure (Amann and Ritz, 1997; Amann *et al.*, 1998, 2000; Nabokov *et al.*, 1999). Using immunohistochemistry, *in situ* hybridization and specific interventions, evidence for a pathophysiological role of the renin–angiotensin (RAS) and the endothelin (ET-1) system has been found (Amann *et al.*, 2000; Nabokov *et al.*, 1999; Wessels *et al*, 1999). In addition, increased expression of growth factors and alteration in the extracellular matrix has been documented (Amann *et al.*, 1998).

It was the aim of the present study to: (a) investigate changes in cardiac gene expression with particular focus on fibrosis in the SNX model at an early (2 weeks) and a late time point (12 weeks), using high-throughput techniques; and (b) to confirm earlier data on activation of the abovementioned pathophysiological pathways.

### Materials and methods

### Animals

Male 200 g Sprague–Dawley rats were housed in single cages at constant room temperature (20 °C) and humidity (75%) under a controlled light–dark cycle. The rats were fed a diet containing 40 g protein and 0.6 g NaCl per 100 g (Altromin Co., Lage/Lippe, Germany). After a 3 day adaptation period, the animals were randomly allotted to subtotal nephrectomy (SNX, $n = 20$) or sham operation (sham, $n = 20$), which were performed as follows. In a first operation the right kidney was removed under general anaesthesia (ketamin, xylazin) and weighed. The sham operation consisted of decapsulation of the kidney, taking special care not to damage the adrenals. After 1 week, the SNX was completed by resecting the lower and upper poles of the meanwhile hypertrophied left kidney. In order to standardize the procedure, a definite amount of cortex corresponding to 75% of the weight of the resected right kidney was removed (Amann and Ritz, 1997; Amann *et al.*, 1998, 2000; Nabokov *et al.*, 1999). The sham operation was performed as described above. This two-step surgical resection of the renal cortex leads to a moderate and very stable course of renal failure with no or only a very mild increase in systolic blood pressure and associated structural cardiovascular alterations, which have been extensively characterized in previous studies (Amann and Ritz, 1997; Amann *et al.*, 1998, 2000; Nabokov *et al.*, 1999).

### Perfusion fixation, RNA isolation, immunohistochemistry and *in situ* hybridization

After 2 and 12 weeks, respectively, the experiment was terminated by retrograde perfusion fixation via the abdominal aorta in 10 SNX and 10 sham animals, as described in detail elsewhere (Amann and Ritz, 1997; Amann *et al.*, 1998, 2000; Nabokov *et al.*, 1999). Five SNX and five sham animals were perfused using ice-cold NaCl for molecular biology purposes; for the remaining animals 3% glutaraldehyde was used as fixative.

Small pieces of the hearts of five animals per group and time point were homogenized, using a dismembrator (Braun Co, Melsungen, Germany). Total RNA was isolated in each case, using the single-step RNA isolation method (Chomczynski and Sacchi, 1987) with Trizol (Invitrogen Co., Germany). Poly(A)$^+$ RNA was isolated from total RNA, using Dynabeads (Dynal Co., Germany) according to the manufacturer's manual. The remaining heart was cut into transverse sections and fixed with formaldehyde or isopentane for morphological, immunohistological and molecular biological investigations. Immunohistochemical investigations were performed using the avidin–biotin method, as described in detail by Amann *et al.* (1998). Non-radioacitve *in situ* hybridization for angiotensinogen, ET-1, renin and TGF-$\beta$ was performed as described in detail elsewhere (Amann *et al.*, 1998, 2000; Nabokov *et al.*, 1999).

### Rat unigene-1 cDNA array

The Rat unigene-1 array contains 27 350 cDNA clones of the Bento Soares clone collection

Copyright © 2003 John Wiley & Sons, Ltd.

(University of Iowa). The cDNA products were PCR-amplified, using M13 forward and reverse standard primers, and spotted onto a $22 \times 22$ cm nylon membrane in a $5 \times 5$ pattern (RZPD Berlin). For details of the methods, see Boer *et al.* (2001). Each $5 \times 5$ field contained 12 genes spotted in duplicate and one PCR fragment representing the *Escherichia coli* kanamycin resistance gene. This spot was used as the 'empty' spot for background subtraction during data analysis (see below). For quality control, M13 forward and reverse primers were end-labelled with $^{33}$P $\gamma$-ATP and hybridized to each membrane to verify that all filters from the same robot run were spotted evenly and completely. After quality control, the membranes were stripped and used for complex hybridizations after about 6 weeks. Only filters from the same robot run containing comparable concentrations of PCR products representing single genes or ESTs were used for hybridization with the two different samples (sham, SNX; see below). The individual experiments — 2w (2 weeks after operation, see below) and 12w (12 weeks after operation, see below) as well as repetitions — were performed with different filter batches.

### Hybridization of global rat arrays and image analysis

The whole hybridization procedure was done according to Boer *et al.* (2001). 500–1000 ng poly(A)$^+$ RNA was reverse transcribed using (dT)18 primer and 33P $\alpha$-dCTP without amplification and purified. The labelled cDNA was hybridized to the rat unigene-1 array. The hybridization solution contained $6\times$ SSC/5 x Denhardt's, with Cot-1 DNA (Invitrogen, Germany) and (dA)40 oligonucleotide for blocking. After exposition of the hybridized membranes, the PhosphorImager screens were scanned (Fuji FLA-3000, 100 µm resolution; Fuji BAS-reader software). The primary image analysis (estimation of nVol grey level values for each individual spot) was done using the ArrayVision software package (Interfocus), which had been adjusted to the $5 \times 5$ array before. The background was corrected locally in each $5 \times 5$ field by subtracting the empty spot signal. Normalization was done via the average signal intensity (without empty spots) on the whole membrane. The ratio of the spot-to-spot comparison was taken for further analysis; those values close to zero (smaller than 0.001) were eliminated. Since each PCR fragment was spotted twice on each membrane, and each hybridization experiment was performed twice, four ratios were entered into our database tool (Access database program, Microsoft) to make an analysis in a non-statistical manner: three out of four ratios (one outlier was accepted) had to show the same tendency to be included in the final list. For each experiment, two independent hybridizations with five animals per time point were performed.

### Results

### Cardiac structural alterations in the rat model of subtotal nephrectomy

We found evidence for activation of fibrotic and hypertrophic pathways at different time points after the induction of renal failure in the rat model of subtotal nephrectomy.

It is well known from several previous studies using exactly the same animal model that after 2 weeks of renal failure no increase in blood pressure and only a moderate increase in left ventricular weight are observed, whereas activation of interstitial cells is already present (Amann *et al.*, 1994). Afterwards, left ventricular weight increases progressively, leading to marked LVH after 8 and 12 weeks (Amann *et al.*, 1998, 2000; Nabokov *et al.*, 1999). The animals do not, however, develop marked hypertension or anaemia, and plasma renin activation is low, which is in contrast to other models of renal failure (Amann *et al.*, 1994, 1997, 1998, 2000, 2001). Treatment of subtotally nephrectomized rats using sympatholytic agents provided some indirect evidence of a potential activation of the sympathetic nervous system; there is no definite data, however (Amann *et al.*, 2001).

At 8 weeks, LVH of renal failure is accompanied by a significant increase in myocyte diameter, activation and expansion of the non-vascular interstitial tissue, a decrease in capillary density and an increase in wall thickness of small intramyocardial arterioles (Figure 1A, B; Amann and Ritz, 1997, 2001; Amann *et al.*, 1997, 1998, 2000; Nabokov *et al.*, 1999). Due to this time dependency of cardiac lesions, we decided to investigate an early (2 weeks) and a late (12 weeks) time point.

Copyright © 2003 John Wiley & Sons, Ltd. 

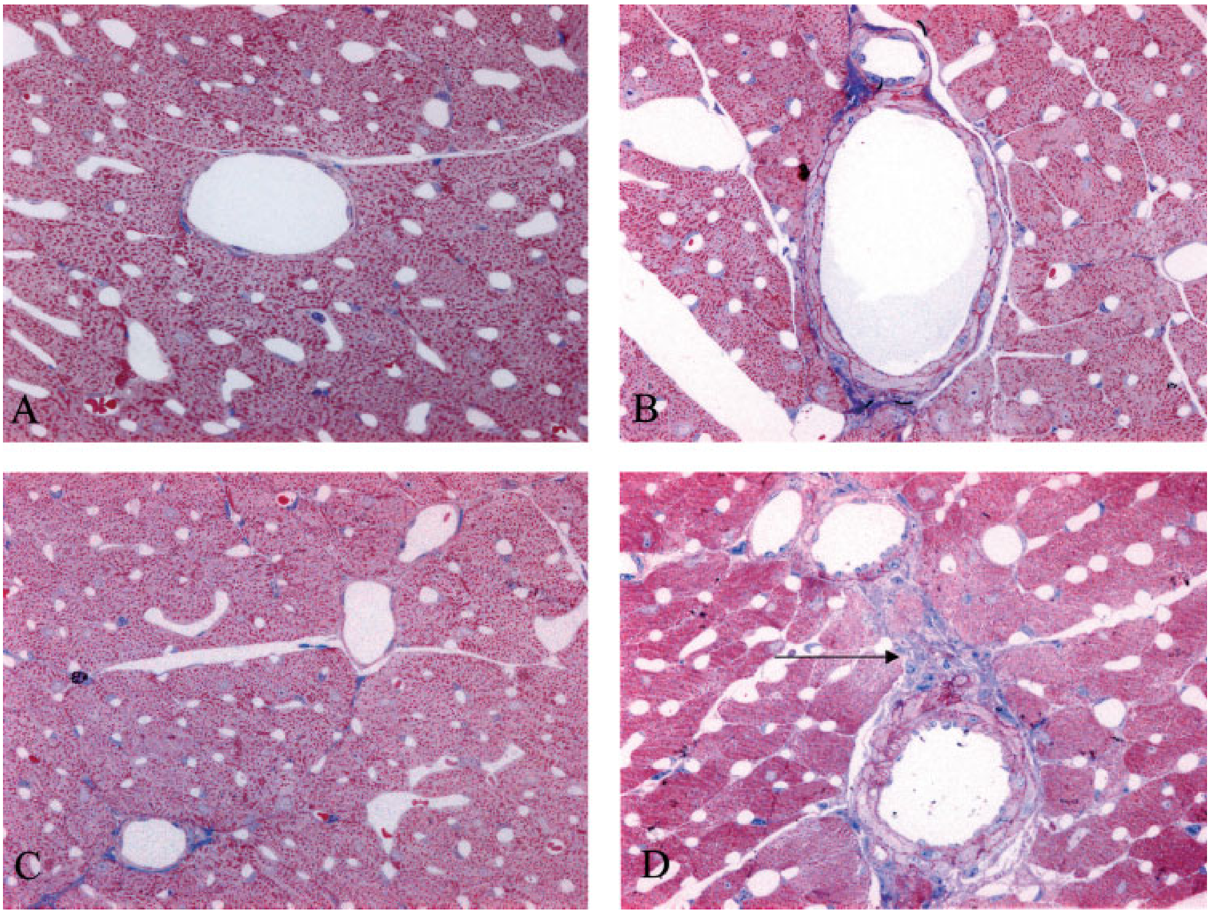

**Figure 1.** Typical morphological changes of the myocardium after 2 (A, B) and 12 weeks (C, D) of renal failure. Hypertrophy of cardiomyocytes, activation of the non-vascular interstitium (arrow) and arteriolar wall thickening is clearly visible in subtotally nephrectomized rats (D) at week 12 compared to sham-operated controls (C)

### Analysis of differentially regulated genes

We were especially interested in extracellular matrix (ECM) genes and in molecules possibly involved in promoting ECM formation. Therefore, for data mining from gene expression profiling experiments, the genes were grouped as follows:

1. Members of the renin–angiotensin system (RAS) as the potentially most important participating hormone system.
2. Members of the group of ECM genes.
3. Genes involved in the regulation of cell junctions (adhesion molecules or structural proteins).
4. Genes involved in cell signalling (G-proteins, *MAP/ERK* cascade, second messenger).
5. Members of the cytoskeleton (structural proteins, motor proteins).
6. Growth factors.

Genes and ESTs belonging to these groups were classified according to the GeneCards database (Weizmann Institute; **http://bioinformatics.weizmann.ac.il/cards/**) and the literature (Figure 2). Altogether, about 400 regulated genes potentially involved in the above-described cardiac lesions could be classified into the selected gene families.

### The renin–angiotensin system (RAS) and downstream pathways are activated in uraemic cardiac hypertrophy

We found an early upregulation of the endothelin B ($ET_B$) receptor in the hypertrophic heart after SNX (Figure 2); this finding was confirmed by *in situ* hybridization, documenting increased

Copyright © 2003 John Wiley & Sons, Ltd. 

ET-1 mRNA in the heart of SNX after 12 w (Figure 3H), as well as an earlier study using PCR (Amann *et al.*, 2000). In addition, using *in situ* hybridization we found increased expression of renin (Wessels *et al.*, 1999) and angiotensinogen RNA in the heart of SNX (Figure 3D) compared to controls (Figure 3C), indicating activation of the local RAS. Moreover, expression of TGF-$\beta$ mRNA was markedly higher in the heart of SNX rats (Figure 3F) compared to controls (Figure 3E).

### G-proteins, second messengers and motor proteins as potential signal mediators

Since the 7-transmembrane receptor $ET_B$ is coupled to further effector systems by nucleotide regulatory proteins (Douglas and Ohlstein, 1997), we focused on this group of proteins in our analysis and also on second messengers as further downstream molecules. We found downregulation of *cdc42* after 2 w, and upregulation of two *RAC* clones (12 w) of *RHO B* (2 w) and ESTs similar to *RHO C* and *RHO A* (12 w). This makes sense, because focal adhesion assembly is mainly mediated by *cdc42, RAC* and members of the Ras superfamily of small GTP-binding proteins and other effectors (Cau *et al.*, 2001; Krendel *et al.*, 2002; Schoenwaelder and Burridge, 1999). C-*JUN*, another signalling molecule, was not differentially regulated in the present model. P38 mitogen-activated protein kinase (*MAPK*), an important member of the cytoplasmic mitogen-activated protein kinase/extracellular-signal regulated kinases (*MAP/ERK*), showed controversial results: One clone was upregulated after 2 w, which would fit to the well known $ET_B$ receptor-dependent activation (Cattaruzza *et al.*, 2001), whereas another clone was downregulated after 2 and 12 w, respectively. A marked regulation of protein kinase C and associated molecules (20 clones), inositol kinases (eight clones), phosphatases (five clones) and phospholipase C (11 clones) points to a major role of these genes in LVH.

There is convincing evidence that motor proteins are involved in the formation of focal adhesions, integrin assembly and ECM formation (Burridge and Chrzanowska-Wodnicka, 1996). We obtained some expected results from our complex hybridizations: 30 myosin clones were differentially regulated, 10 clones (four of them encoding myosin light chains) downregulated after 2 w, 13 clones upregulated and eight clones downregulated after 12 w. Of the 12 dynein clones, three went down after 2 w and four after 12 w (three of them are ESTs), whereas five were upregulated after 12 w. With kinesin, the result was as follows: two clones (kinesin-related protein) down in 2 w, eight clones up in 12 w, two clones down in 12 w. The predominant upregulation of some of the cytoskeleton-related proteins at 12 w might be initiated by LIM proteins (Khurana *et al.*, 2002).

### The extracellular matrix (ECM) accumulates during cardiac remodelling

As a central linker protein between the cytoplasm and the extracellular matrix, we found integrin-$\beta$1 to be upregulated, especially in the 12 w sample (Figure 2). This result was confirmed by immunohistochemistry (Figure 3A, B; Amann *et al.*, 1998). 28 clones of the 128 ECM-linked clones listed in Figure 1 code for collagen subunits or enzymes involved in collagen turnover. Most genes were upregulated after 2 w, and no further change was detected after 12 w. However, single clones were upregulated after 12 w, one being a collagenase (UMCase). This indicates that collagen turnover continues at a somewhat lower level after 2 w. Two procollagens even appear to be downregulated after 2 and 12 w, respectively, whereas another clone (procollagen C-proteinase enhancer protein, PCOLCE) was upregulated after 2 and 12 w. A number of proteoglycans, another important group involved in ECM metabolism, were also upregulated as early as 2 w after SNX. Several SPARC clones were downregulated, which is in agreement with other findings (Schoenwaelder and Burridge, 1999). Other mediators of ECM formation found to be upregulated were tissue inhibitor of metalloproteinase 3 (TIMP3) at 2 w and 12 w, matrix metalloproteinase 7 (MMP7) at 2 w, laminins (eight clones) at 12 w, plectin (three clones) at 12 w, cell adhesion kinase $\beta$ at 12 w, and N-cadherins and catenins.

### Discussion

Renal failure is associated with complex cardiac remodelling that occurs very early on in the course of the disease (Parfrey *et al.*, 1996; Stefanski *et al.*, 1996) and it is characterized by LVH with accompanying changes in the myocardial composition,

Copyright © 2003 John Wiley & Sons, Ltd. 

| 2W | 12W | Name | RZPD ID |
|---|---|---|---|
| **renin angiotensin system** | | | |
| 0.3 | 2.3 | angiotensin receptor 2 | UI__p953M2156 |
| | 0.6 | angiotensin II AT2 receptor-interacting protein (EST) | UI__p953J1150 |
| 2.2 | | endothelin-converting enzyme | UI__p953C052 |
| 0.6 | 0.9 | endothelin-converting enzyme | UI__p953L039 |
| 3.9 | | endothelin receptor | UI__p953E0838 |
| **second messengers, G-proteins, MAP/ERK cascade** | | | |
| 1.7 | | MAP-kinase phosphatase (cpg21) | UI__p953H0342 |
| 0.7 | 0.9 | MAP kinase kinase 7 beta 2 (EST) | UI__p953B1155 |
| 0.5 | 1.5 | MAP kinase kinase 5 | UI__p953C1945 |
| 0.6 | | MAP kinase activated protein kinase 2 (EST) | UI__p953H0910 |
| 0.6 | 0.6 | MAP kinase activated protein kinase 2 (EST) | UI__p953O1730 |
| | 0.7 | MAP kinase kinase mRNA | UI__p953E1312 |
| 1.1 | | myo-inositol monophosphatase (IMP) | UI__p953K049 |
| 1.9 | 3.6 | myo-inositol monophosphatase (IMP) | UI__p953K2269 |
| | 2.7 | myo-inositol monophosphatase (IMP) | UI__p953D111 |
| | 0.7 | myo-inositol monophosphatase (IMP) | UI__p953K049 |
| 0.8 | 0.6 | myo-inositol 1-phosphate synthase (EST) | UI__p953N0628 |
| | 0.8 | myo-inositol 1-phosphate synthase (EST) | UI__p953K1016 |
| | 2.6 | myo-inositol 1-phosphate synthase (EST) | UI__p953I032 |
| 3.1 | 0.5 | myo-inositol-1(or 4) monophosphatase (EST) | UI__p953D179 |
| 0.9 | | diphosphoinositol polyphosp. Phosphohydrol. (EST) | UI__p953D1817 |
| 0.2 | | phosphatidylinositol synthase | UI__p953F0416 |
| 0.6 | | phosphatidylinositol 3-kinase (EST) | UI__p953I054 |
| 0.5 | | phosphatidylinositol 4-kinase (EST) | UI__p953J1471 |
| | 4.6 | phosphatidylinositol glycan class F (EST) | UI__p953B0618 |
| | 1.8 | phosphatidylinositol 4-phosph. 5-kin.alpha type1 (EST) | UI__p953K0657 |
| | 3.5 | phosphatidylinositol 5-phosph. 4-kin. gamma (EST) | UI__p953K1914 |
| 2.4 | 0.7 | phosphatidylinositol 4-kinase (EST) | UI__p953E233 |
| 2.2 | 0.4 | phosphatidylinositol 3-kinase (EST) | UI__p953F0948 |
| 1.3 | | phosphatidylinositol 4-kinase | UI__p953G1310 |
| | 3.8 | inositol 1, 4, 5-triphosphate receptor 3 | UI__p953M0617 |
| | 0.1 | inositol 1, 4, 5-triphosphate receptor 3 | UI__p953H2410 |
| | 0.5 | inositol 1, 4, 5-triphosphate receptor 1 | UI__p953N1156 |
| 1.5 | | inositol hexakisphosphate kinase, complete cds | UI__p953I218 |
| 2.7 | | inositol polyphosphate 1 phosphatase (EST) | UI__p953I2370 |
| 3.6 | | inositol polyphosphate 5-phosphat. (PIPP, proline-rich) | UI__p953K0760 |
| 0.6 | | inositol 1,4,5-trisphosph.bind.prot.type1 rec. (EST) | UI__p953B153 |
| | 0.6 | multiple inosit.polyphosph.histidinephosphat.1 (EST) | UI__p953C173 |
| | 0.3 | inositol polyphosphate 5-phosphatase (EST) | UI__p953J0451 |
| 2.2 | 1.4 | phospholipase A2 | UI__p953I2117 |
| 0.4 | | phospholipase A2 (Ca-independent, EST) | UI__p953K2441 |
| 2.0 | | phospholipase C (EST) | UI__p953A1566 |
| | 1.7 | phospholipase C (EST) | UI__p953E2244 |
| 2.0 | | phospholipase C (EST) | UI__p953H1442 |
| 1.4 | | phospholipase C (EST) | UI__p953L0430 |
| 3.1 | | phospholipase C | UI__p953K1548 |
| | 2.1 | phospholipase C gamma 1 | UI__p953P0429 |
| 0.5 | 0.8 | phospholipase C gamma 1 | UI__p953D183 |
| | 0.8 | phospholipase C (EST) | UI__p953A1727 |
| 0.7 | | Rattus norvegicus phospholipase C delta 4 (EST) | UI__p953G1135 |
| 0.1 | | Phospholipase C delta1 | UI__p953I0240 |
| | 0.7 | phospholipase C (EST) | UI__p953C239 |
| 1.3 | 1.5 | PKCq-interacting protein PICOT | UI__p953J1314 |
| | 1.9 | PKC zeta interacting protein (EST) | UI__p953A2454 |
| | 1.4 | PKC zeta | UI__p953H11 |
| 3.4 | 5.2 | PKC zeta interacting protein | UI__p953L1746 |
| 1.2 | 0.7 | PKC zeta interacting protein (EST) | UI__p953M1768 |
| 0.7 | | PKC zeta interacting protein (EST) | UI__p953B1010 |
| 0.7 | | PKC zeta interacting protein (EST) | UI__p953L028 |
| 1.3 | 2.8 | protein kinase C receptor | UI__p953B1148 |
| 2.4 | | protein kinase C receptor | UI__p953H092 |
| 1.4 | | protein kinase C receptor | UI__p953J152 |
| 1.6 | | protein kinase C substrate | UI__p953L1048 |
| 2.1 | | protein kinase C substrate (EST) | UI__p953E1713 |
| 3.7 | | protein kinase C binding protein Enigma | UI__p953L2470 |
| 0.8 | 0.7 | protein kinase C delta binding protein | UI__p953K2218 |
| 0.3 | 1.6 | protein kinase C substrate | UI__p953N1727 |
| 0.6 | | protein kinase C epsilon (EST) | UI__p953N1754 |
| 0.7 | | protein kinase C binding protein Enigma (EST) | UI__p953O2435 |
| | 1.4 | protein kinase C-binding protein NELL2 | UI__p953E0520 |
| | 1.7 | protein kinase C-binding protein Beta15 | UI__p953I1034 |
| | 0.2 | protein kinase C gamma (EST) | UI__p953A0745 |
| | 3.0 | calcium/calmodulin-dependent serine protein kinase | UI__p953J2249 |
| 2.0 | 1.4 | adenylate cyclase activating polypeptide 1 | UI__p953A0266 |
| 0.5 | | adenylate cyclase activating polypeptide 1 | UI__p953P1736 |
| 1.4 | 2.9 | guanylate cyclase alpha 1 | UI__p953J1526 |
| | 1.3 | guanylate cyclase A receptor A | UI__p953J1949 |
| | 0.5 | guanylate cyclase | UI__p953D0939 |
| 1.8 | | rhoB gene | UI__p953B098 |
| 3.4 | | Rho-associated kinase beta mRNA (EST) | UI__p953M0870 |
| 0.3 | 2.5 | Rho-guanine nucleotide exchange factor (EST) | UI__p953N245 |
| 0.7 | 2.6 | RhoC | UI__p953O2317 |
| | 2.5 | RhoA (EST) | UI__p953B0567 |
| | 0.6 | RhoA (EST) | UI__p953F2412 |
| | 0.5 | Rho (EST) | UI__p953F0570 |
| | 0.6 | RhoC (EST) | UI__p953H193 |
| | 2.4 | protein kinase alpha (RAC) | UI__p953J1067 |
| | 2.9 | protein kinase alpha (RAC) | UI__p953M0327 |
| 5.7 | | c-Jun leucine zipper interactive (EST) | UI__p953D2271 |
| 0.6 | 1.5 | c-Jun leucine zipper interactive (EST) | UI__p953H0133 |
| | 0.9 | c-Jun leucine zipper interactive (EST) | UI__p953F2333 |
| 0.6 | | dystrophy kinase-relatedCdc42-bindingkinase(MRCK) | UI__p953G2351 |
| 5.0 | | cdc42 associated protein (GTP hydrolysis, EST) | UI__p953D036 |
| 0.6 | | cell division cycle 42 (cdc42) | UI__p953G2451 |
| **cytoskeleton** | | | |
| 1.7 | | LIM protein (alpha actinin associated) | UI__p953A0714 |
| 1.7 | 1.9 | LIM protein | UI__p953B1415 |
| 1.8 | | LIM protein (EST) | UI__p953B2067 |
| 1.3 | 0.8 | LIM protein (reversion induced) | UI__p953C2411 |
| 1.8 | | LIM protein (EST) | UI__p953J0726 |
| 2.3 | 0.5 | LIM protein (SmLIM) | UI__p953N0217 |
| 0.6 | 0.6 | LIM protein (alpha actinin associated) | UI__p953C2038 |
| 0.6 | | LIM protein | UI__p953I1421 |
| 0.7 | | LIM binding factor | UI__p953L0951 |
| | 1.7 | beta-actin | UI__p953H2152 |
| | 2.0 | beta-actin | UI__p953J2028 |
| | 1.7 | beta-actin (EST) | UI__p953N1316 |
| | 1.5 | alpha-actin (EST) | UI__p953L048 |
| | 1.3 | actin-capping protein beta chain (EST) | UI__p953H0210 |
| | 1.4 | actin-related protein 3 (Arp3, EST) | UI__p953B114 |
| | 3.3 | actin single-stranded DNA-binding factor 2 p44 (EST) | UI__p953B1861 |
| | 4.0 | microtubule-actin crosslinking factor (EST) | UI__p953G098 |
| | 1.8 | microtubule-actin crosslinking factor (EST) | UI__p953C1526 |
| | 1.2 | actin binding protein 1B (coronin) | UI__p953D029 |
| 1.2 | | actin alpha (PKC interacting, EST) | UI__p953L048 |
| | 2.8 | alpha-actinin 4 | UI__p953D1870 |
| | 1.3 | F-actin binding protein b-Nexilin | UI__p953J2340 |
| 0.5 | 0.9 | beta-actin (EST) | UI__p953C123 |
| 0.7 | | microtubule-actin crosslinking factor (EST) | UI__p953G098 |
| | 0.6 | actin-related protein complex 1b | UI__p953M228 |
| | 0.7 | actin-related protein complex 1b | UI__p953B1417 |
| 2.2 | 0.6 | actin single-stranded DNA-binding factor 2 p44 (EST) | UI__p953J1628 |
| 0.8 | 0.7 | cofilin 1 (actin-associated) | UI__p953J2129 |
| 1.8 | | cofilin 1 (actin-associated) | UI__p953E0828 |
| 0.3 | | entactin-2 (EST) | UI__p953O075 |
| | 0.7 | decorin (DCN) | UI__p953H2266 |
| | 0.7 | dynactin (p62, EST) | UI__p953D233 |
| | 3.4 | dynactin (p62, EST) | UI__p953G203 |
| | 1.6 | dynactin (p62, EST) | UI__p953I1211 |
| 0.7 | 1.5 | dynactin (DYNC, 50 kd isoform, EST) | UI__p953J192 |
| 0.8 | 1.2 | dynactin (DYNC, 50 kd isoform, EST) | UI__p953F1847 |
| | 1.6 | dynactin (DYNC, 150 kd isoform, EST) | UI__p953B1614 |
| 0.4 | | dynactin (p62, EST) | UI__p953N0929 |
| | 1.8 | dynein light chain 1 (EST) | UI__p953J052 |
| | 0.7 | dynein light chain 1 (EST) | UI__p953A1551 |
| | 0.8 | dynein light intermediate chain 1 | UI__p953L032 |
| 0.8 | 1.2 | dynein light intermediate chain 1 | UI__p953G128 |
| 0.8 | 0.7 | dynein intermediate chain 2C (cytoplasmic) | UI__p953I0136 |
| | 0.8 | dynein heavy chain (EST) | UI__p953M224 |
| | 2.6 | dynein heavy chain (EST) | UI__p953E0439 |
| | 3.8 | dynein heavy chain (EST) | UI__p953G204 |
| | 4.3 | dynein light intermediate chain 53/55 | UI__p953F0726 |
| 0.6 | | dynein-associated polypeptide DP150 | UI__p953L1216 |
| | 2.2 | entactin 2 (EST) | UI__p953O075 |
| | 0.6 | filamin gamma (EST) | UI__p953C0511 |
| | 3.6 | keratin K2C8 (EST) | UI__p953L108 |
| | 2.9 | keratin 1 48 kd (EST) | UI__p953K223 |
| | 1.5 | keratin 1 48 kd (EST) | UI__p953A1210 |
| 0.7 | | keratin-like protein (EST) | UI__p953J1910 |
| | 0.3 | cytokeratin 18 | UI__p953M015 |
| | 3.2 | kinesin light chain C | UI__p953D0929 |
| | 2.2 | kinesin light chain A (EST) | UI__p953E024 |
| | 1.6 | kinesin heavy chain (EST) | UI__p953D1712 |
| | 2.3 | kinesin related protein (EST) | UI__p953G0671 |
| 0.8 | 2.1 | kinesin related protein (EST) | UI__p953D025 |
| 0.8 | 1.7 | kinesin related protein (EST) | UI__953J1822 |
| | 1.3 | kinesin like protein (EST) | UI__p953C2063 |
| | 2.8 | rabkinesin-6 (EST) | UI__p953L1447 |
| 1.5 | 1.3 | kinesin like protein (KF1A, EST) | UI__p953E213 |
| | 0.6 | kinesin like protein (KIF1D, EST) | UI__p953J1948 |
| | 0.5 | kinesin like protein (KIF1B, EST) | UI__p953K0866 |
| | 0.7 | myosin regulatory light chain 2 (EST) | UI__p953N1323 |
| | 0.8 | myosin heavy chain | UI__p953J024 |
| | 0.9 | myosin light chain 2 (MLC2) | UI__p953A159 |
| | 0.6 | myosin regulatory light chain | UI__p953M2110 |
| | 0.4 | myosin heavy chain | UI__p953O2423 |
| 0.8 | 0.8 | myosin light chain (alkali, EST) | UI__p953C0730 |
| 0.3 | | myosin light chain (alkali EST) | UI__p953L063 |
| 0.6 | | myosin light chain | UI__p953F1955 |
| 0.4 | | myosin (unconventional) | UI__p953A072 |
| 0.5 | | myosin I heavy chain | UI__p953O0714 |
| 0.8 | 2.1 | myosin I heavy chain (MYSB, EST) | UI__p953P105 |
| 0.6 | | myosin like protein X (EST) | UI__p953H1046 |
| | 0.5 | myosin like protein X (EST) | UI__p953M2348 |
| 0.3 | | myosin-RhoGAP protein Myr7 | UI__p953K1752 |
| | 0.3 | myosin-RhoGAP protein Myr7 (EST) | UI__p953N2469 |
| 0.4 | | myosin regulatory light chain 2 | UI__p953N1323 |
| | 2.9 | myosin heavy chain 3 | UI__p953M0667 |
| | 2.8 | myosin heavy chain | UI__p953E0713 |
| | 1.5 | myosin heavy chain | UI__p953F158 |
| | 2.1 | myosin heavy chain B (plasmolipin) | UI__p953E133 |
| | 1.4 | myosin light chain 1(EST) | UI__p953O0760 |
| | 1.6 | myosin light chain 1(EST) | UI__p953P0217 |
| | 2.0 | myosin light chain 3 | UI__p953D0241 |
| | 1.5 | myosin light chain 3 | UI__p953N074 |
| 0.6 | 2.6 | myosin heavy chain | UI__p953I071 |
| | 2.0 | myosin heavy chain | UI__p953D122 |
| | 1.6 | myosin I heavy chain (MYR2) | UI__p953I1811 |
| | 2.5 | myosin I heavy chain (unconventional) | UI__p953E1211 |
| | 1.2 | myosin regulatory light chain | UI__p953D0671 |
| | 1.7 | plectin | UI__p953G1017 |
| 0.6 | 1.5 | plectin (EST) | UI__p953K1522 |
| | 1.3 | plectin (EST) | UI__p953B1154 |

**Figure 2.** Summary of differentially regulated genes belonging to gene families investigated in this study. Red, clones upregulated in SNX vs. sham (2 weeks/12 weeks); green, clones downregulated in SNX vs. sham (2 weeks/12 weeks). The gene names are given according to the UniGene database. Further information concerning the listed genes is accessible with the RZPD clone ID via the RZPD database (**www.rzpd.de**) or other databases

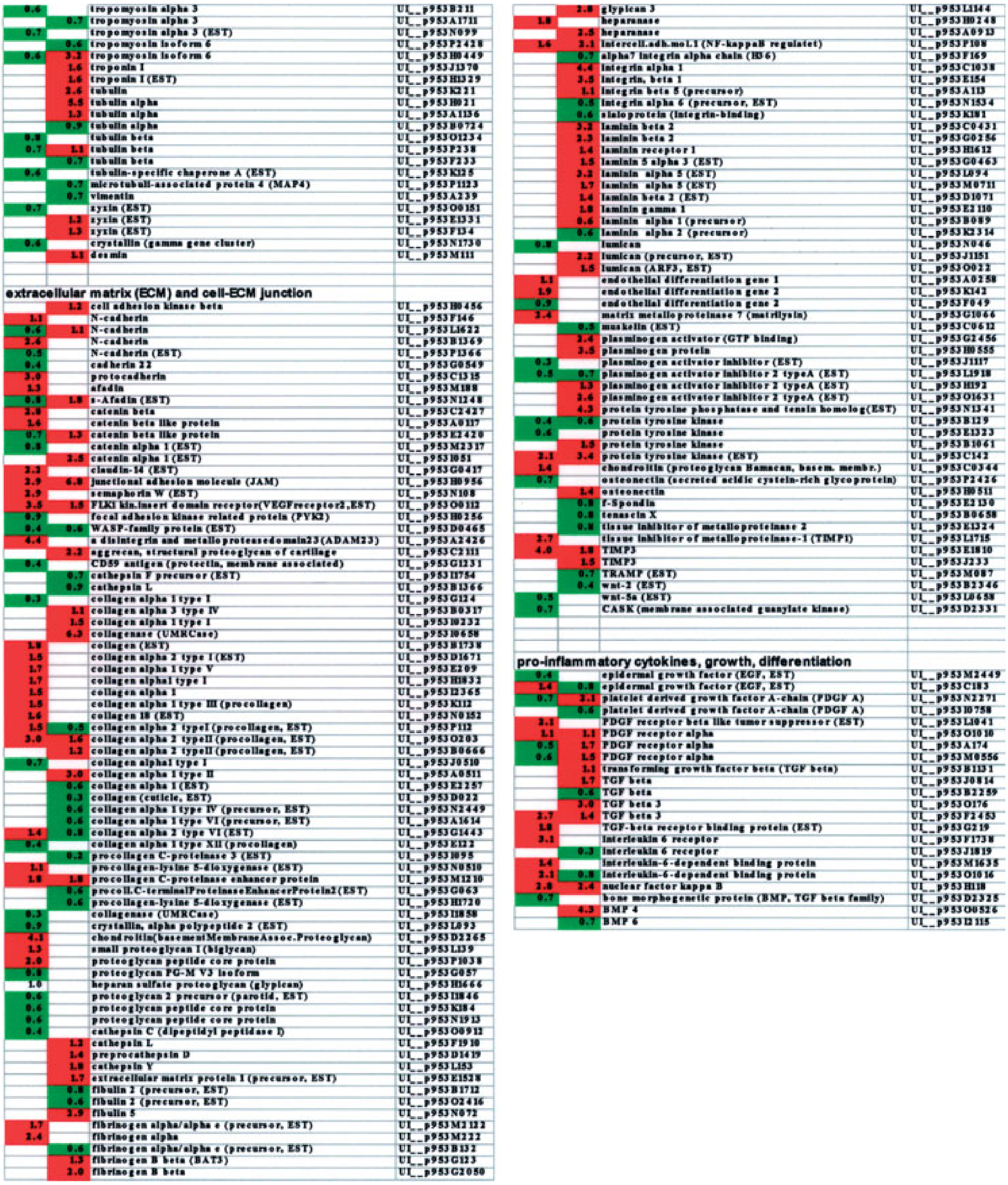

| | | | |
|---|---|---|---|
| 0.6 | | tropomyosin alpha 3 | UI__p953B211 |
| | 0.7 | tropomyosin alpha 3 | UI__p953A1711 |
| 0.7 | | tropomyosin alpha 3 (EST) | UI__p953N099 |
| | 0.6 | tropomyosin isoform 6 | UI__p953P2428 |
| 0.6 | 3.2 | tropomyosin isoform 6 | UI__p953H0449 |
| | 1.6 | troponin I | UI__p953J1370 |
| | 1.6 | troponin I (EST) | UI__p953H1329 |
| | 2.6 | tubulin | UI__p953K221 |
| | 5.5 | tubulin alpha | UI__p953H021 |
| | 1.3 | tubulin alpha | UI__p953A1136 |
| | 0.9 | tubulin alpha | UI__p953B0724 |
| 0.8 | | tubulin beta | UI__p953O1234 |
| 0.7 | 1.1 | tubulin beta | UI__p953P238 |
| | 0.7 | tubulin beta | UI__p953F233 |
| 0.6 | | tubulin-specific chaperone A (EST) | UI__p953K125 |
| | 0.7 | microtubuli-associated protein 4 (MAP4) | UI__p953P1123 |
| | 0.7 | vimentin | UI__p953A239 |
| 0.7 | | zyxin (EST) | UI__p953O0151 |
| | 1.2 | zyxin (EST) | UI__p953E1331 |
| | 1.3 | zyxin (EST) | UI__p953F134 |
| 0.6 | | crystallin (gamma gene cluster) | UI__p953N1730 |
| | 1.1 | desmin | UI__p953M111 |
| | | | |
| | | | |
| **extracellular matrix (ECM) and cell-ECM junction** | | | |
| | 1.2 | cell adhesion kinase beta | UI__p953H0456 |
| 1.1 | | N-cadherin | UI__p953F146 |
| 0.6 | 1.1 | N-cadherin | UI__p953L1622 |
| 2.6 | | N-cadherin | UI__p953B1369 |
| 0.5 | | N-cadherin (EST) | UI__p953P1366 |
| 0.4 | | cadherin 22 | UI__p953G0549 |
| 3.0 | | protocadherin | UI__p953C1315 |
| 1.3 | | afadin | UI__p953M188 |
| 0.8 | 1.8 | s-Afadin (EST) | UI__p953N1248 |
| 2.8 | | catenin beta | UI__p953C2427 |
| 1.6 | | catenin beta like protein | UI__p953A0117 |
| 0.7 | 1.3 | catenin beta like protein | UI__p953E2420 |
| 0.8 | | catenin alpha 1 (EST) | UI__p953M2317 |
| | 2.5 | catenin alpha 1 (EST) | UI__p953I051 |
| 2.2 | | claudin-14 (EST) | UI__p953G0417 |
| 2.9 | 6.8 | junctional adhesion molecule (JAM) | UI__p953H0956 |
| 2.9 | | semaphorin W (EST) | UI__p953N108 |
| 3.5 | 1.5 | FLK1 kin.insert domain receptor(VEGFreceptor2,EST) | UI__p953O0112 |
| 0.9 | | focal adhesion kinase related protein (PYK2) | UI__p953H0256 |
| 0.4 | 0.6 | WASP-family protein (EST) | UI__p953D0465 |
| 4.4 | | a disintegrin and metalloproteasedomain23(ADAM23) | UI__p953A2426 |
| | 2.2 | aggrecan, structural proteoglycan of cartilage | UI__p953C2111 |
| 0.4 | | CD59 antigen (protectin, membrane associated) | UI__p953G1231 |
| | 0.7 | cathepsin F precursor (EST) | UI__p953I1754 |
| | 0.9 | cathepsin L | UI__p953B1366 |
| 0.3 | | collagen alpha 1 type I | UI__p953G124 |
| | 1.1 | collagen alpha 3 type IV | UI__p953B0317 |
| | 1.5 | collagen alpha 1 type I | UI__p953I0232 |
| | 6.3 | collagenase (UMRCase) | UI__p953I0658 |
| 1.8 | | collagen (EST) | UI__p953B1738 |
| 1.5 | | collagen alpha 2 type I (EST) | UI__p953D1671 |
| 1.7 | | collagen alpha 1 type V | UI__p953E209 |
| 1.7 | | collagen alpha1 type I | UI__p953H1832 |
| 1.5 | | collagen alpha 1 | UI__p953I2365 |
| 1.5 | | collagen alpha 1 type III (procollagen) | UI__p953K112 |
| 1.6 | | collagen 18 (EST) | UI__p953N0152 |
| 1.5 | 0.5 | collagen alpha 2 typeI (procollagen, EST) | UI__p953P112 |
| 3.0 | 1.6 | collagen alpha 2 typeII (procollagen, EST) | UI__p953O203 |
| | 1.2 | collagen alpha 2 typeII (procollagen, EST) | UI__p953B0666 |
| 0.7 | | collagen alpha1 type I | UI__p953J0510 |
| | 3.0 | collagen alpha 1 type II | UI__p953A0511 |
| | 0.6 | collagen alpha 1 (EST) | UI__p953E2257 |
| | 0.3 | collagen (cuticle, EST) | UI__p953D022 |
| | 0.6 | collagen alpha 1 type IV (precursor, EST) | UI__p953N2449 |
| | 0.6 | collagen alpha 1 type VI (precursor, EST) | UI__p953A1614 |
| 1.4 | 0.8 | collagen alpha 2 type VI (EST) | UI__p953G1443 |
| 0.4 | | collagen alpha 1 type XII (procollagen) | UI__p953E122 |
| | 0.2 | procollagen C-proteinase 3 (EST) | UI__p953I095 |
| 1.1 | | procollagen-lysine 5-dioxygenase (EST) | UI__p953N0510 |
| 1.8 | 1.8 | procollagen C-proteinase enhancer protein | UI__p953M1210 |
| | 0.6 | procoll.C-terminalProteinaseEnhancerProtein2(EST) | UI__p953G063 |
| | 0.6 | procollagen-lysine 5-dioxygenase (EST) | UI__p953H1720 |
| 0.3 | | collagenase (UMRCase) | UI__p953I1858 |
| 0.9 | | crystallin, alpha polypeptide 2 (EST) | UI__p953L093 |
| 4.1 | | chondroitin(basementMembraneAssoc.Proteoglycan) | UI__p953D2265 |
| 1.3 | | small proteoglycan I (biglycan) | UI__p953L139 |
| 2.0 | | proteoglycan peptide core protein | UI__p953P1038 |
| 0.8 | | proteoglycan PG-M V3 isoform | UI__p953G057 |
| 1.0 | | heparan sulfate proteoglycan (glypican) | UI__p953H1666 |
| 0.6 | | proteoglycan 2 precursor (parotid, EST) | UI__p953I1846 |
| 0.6 | | proteoglycan peptide core protein | UI__p953K184 |
| 0.6 | | proteoglycan peptide core protein | UI__p953N1913 |
| 0.4 | | cathepsin C (dipeptidyl peptidase I) | UI__p953O0912 |
| | 1.2 | cathepsin L | UI__p953F1910 |
| | 1.4 | preprocathepsin D | UI__p953D1419 |
| | 1.8 | cathepsin Y | UI__p953L153 |
| | 1.7 | extracellular matrix protein 1 (precursor, EST) | UI__p953E1528 |
| | 0.8 | fibulin 2 (precursor, EST) | UI__p953B1712 |
| | 0.6 | fibulin 2 (precursor, EST) | UI__p953O2416 |
| | 2.9 | fibulin 5 | UI__p953N072 |
| 1.7 | | fibrinogen alpha/alpha e (precursor, EST) | UI__p953M2122 |
| 2.4 | | fibrinogen alpha | UI__p953M222 |
| | 0.6 | fibrinogen alpha/alpha e (precursor, EST) | UI__p953B132 |
| | 1.3 | fibrinogen B beta (BAT3) | UI__p953G123 |
| | 2.0 | fibrinogen B beta | UI__p953G2050 |

| | | | |
|---|---|---|---|
| | 2.8 | glypican 3 | UI__p953L1144 |
| 1.8 | | heparanase | UI__p953H0248 |
| | 2.5 | heparanase | UI__p953A0913 |
| 1.6 | 2.1 | intercell.adh.mol.1 (NF-kappaB regulated) | UI__p953P108 |
| | 0.7 | alpha7 integrin alpha chain (H36) | UI__p953F169 |
| | 4.4 | integrin alpha 1 | UI__p953C1038 |
| | 3.5 | integrin, beta 1 | UI__p953E154 |
| | 1.1 | integrin beta 5 (precursor) | UI__p953A113 |
| | 0.5 | integrin alpha 6 (precursor, EST) | UI__p953N1534 |
| | 0.6 | sialoprotein (integrin-binding) | UI__p953K181 |
| | 3.2 | laminin beta 2 | UI__p953C0431 |
| | 2.3 | laminin beta 2 | UI__p953G0256 |
| | 1.4 | laminin receptor 1 | UI__p953H1612 |
| | 1.5 | laminin 5 alpha 3 (EST) | UI__p953G0463 |
| | 3.2 | laminin alpha 5 (EST) | UI__p953L094 |
| | 1.7 | laminin alpha 5 (EST) | UI__p953M0711 |
| | 1.4 | laminin beta 2 (EST) | UI__p953D1071 |
| | 1.8 | laminin gamma 1 | UI__p953E2110 |
| | 0.6 | laminin alpha 1 (precursor) | UI__p953B089 |
| | 0.6 | laminin alpha 2 (precursor) | UI__p953K2314 |
| 0.8 | | lumican | UI__p953N046 |
| | 2.2 | lumican (precursor, EST) | UI__p953J1151 |
| | 1.5 | lumican (ARF3, EST) | UI__p953O022 |
| 1.1 | | endothelial differentiation gene 1 | UI__p953A0258 |
| 1.9 | | endothelial differentiation gene 2 | UI__p953K142 |
| 0.9 | | endothelial differentiation gene 2 | UI__p953F049 |
| 3.4 | | matrix metalloproteinase 7 (matrilysin) | UI__p953G1066 |
| | 0.5 | muskelin (EST) | UI__p953C0612 |
| | 2.4 | plasminogen activator (GTP binding) | UI__p953G2456 |
| | 3.5 | plasminogen protein | UI__p953H0555 |
| 0.3 | | plasminogen activator inhibitor (EST) | UI__p953J1117 |
| 0.5 | 0.7 | plasminogen activator inhibitor 2 typeA (EST) | UI__p953L1918 |
| | 1.3 | plasminogen activator inhibitor 2 typeA (EST) | UI__p953H192 |
| | 2.6 | plasminogen activator inhibitor 2 typeA (EST) | UI__p953O1631 |
| | 4.3 | protein tyrosine phosphatase and tensin homolog(EST) | UI__p953N1341 |
| 0.4 | 0.6 | protein tyrosine kinase | UI__p953B129 |
| 0.6 | | protein tyrosine kinase | UI__p953E1323 |
| | 1.5 | protein tyrosine kinase | UI__p953B1061 |
| 2.1 | 3.4 | protein tyrosine kinase (EST) | UI__p953C142 |
| 1.4 | | chondroitin (proteoglycan Bamacan, basem. membr.) | UI__p953C0344 |
| 0.7 | | osteonectin (secreted acidic cystein-rich glycoprotein) | UI__p953P2426 |
| | 1.4 | osteonectin | UI__p953H0511 |
| | 0.5 | f-Spondin | UI__p953E2130 |
| | 0.8 | tenascin X | UI__p953B0658 |
| | 0.8 | tissue inhibitor of metalloproteinase 2 | UI__p953E1324 |
| 2.7 | | tissue inhibitor of metalloproteinase-1 (TIMP1) | UI__p953L1715 |
| 4.0 | 1.8 | TIMP3 | UI__p953E1810 |
| | 1.5 | TIMP3 | UI__p953J233 |
| | 0.7 | TRAMP (EST) | UI__p953M087 |
| | 0.4 | wnt-2 (EST) | UI__p953B2346 |
| 0.5 | | wnt-5a (EST) | UI__p953L0658 |
| 0.7 | | CASK (membrane associated guanylate kinase) | UI__p953D2331 |
| | | | |
| | | | |
| **pro-inflammatory cytokines, growth, differentiation** | | | |
| 0.4 | | epidermal growth factor (EGF, EST) | UI__p953M2449 |
| 1.4 | 0.8 | epidermal growth factor (EGF, EST) | UI__p953C183 |
| 0.7 | 2.1 | platelet derived growth factor A-chain (PDGF A) | UI__p953N2271 |
| | 0.6 | platelet derived growth factor A-chain (PDGF A) | UI__p953I0758 |
| 2.1 | | PDGF receptor beta like tumor suppressor (EST) | UI__p953L1041 |
| 1.1 | 1.1 | PDGF receptor alpha | UI__p953O1010 |
| 0.5 | 1.7 | PDGF receptor alpha | UI__p953A174 |
| 0.6 | 1.5 | PDGF receptor alpha | UI__p953M0556 |
| | 1.1 | transforming growth factor beta (TGF beta) | UI__p953B1131 |
| | 1.7 | TGF beta | UI__p953J0814 |
| | 0.6 | TGF beta | UI__p953B2259 |
| | 3.0 | TGF beta 3 | UI__p953O176 |
| 2.7 | 1.4 | TGF beta 3 | UI__p953F2453 |
| 1.8 | | TGF-beta receptor binding protein (EST) | UI__p953G219 |
| 3.1 | | interleukin 6 receptor | UI__p953F1738 |
| | 0.3 | interleukin 6 receptor | UI__p953J1819 |
| 1.4 | | interleukin-6-dependent binding protein | UI__p953M1635 |
| 2.1 | 0.8 | interleukin-6-dependent binding protein | UI__p953O1016 |
| 2.8 | 2.4 | nuclear factor kappa B | UI__p953H118 |
| 0.7 | | bone morphogenetic protein (BMP, TGF beta family) | UI__p953D2325 |
| | 4.3 | BMP 4 | UI__p953O0526 |
| | 0.7 | BMP 6 | UI__p953I2115 |

**Figure 2.** Continued i.e. interstitial fibrosis and impaired microcirculation. Activation and expansion of cardiac interstitial tissue is an important hallmark of cardiac remodelling in uraemic patients and in rats with renal failure (Amann and Ritz, 1997; Amann *et al*., 1994; Mall *et al*., 1990). Using electron microscopy, activation and enlargement of interstitial fibroblasts was seen as early as 2 weeks after SNX (Amann

Copyright © 2003 John Wiley & Sons, Ltd. 

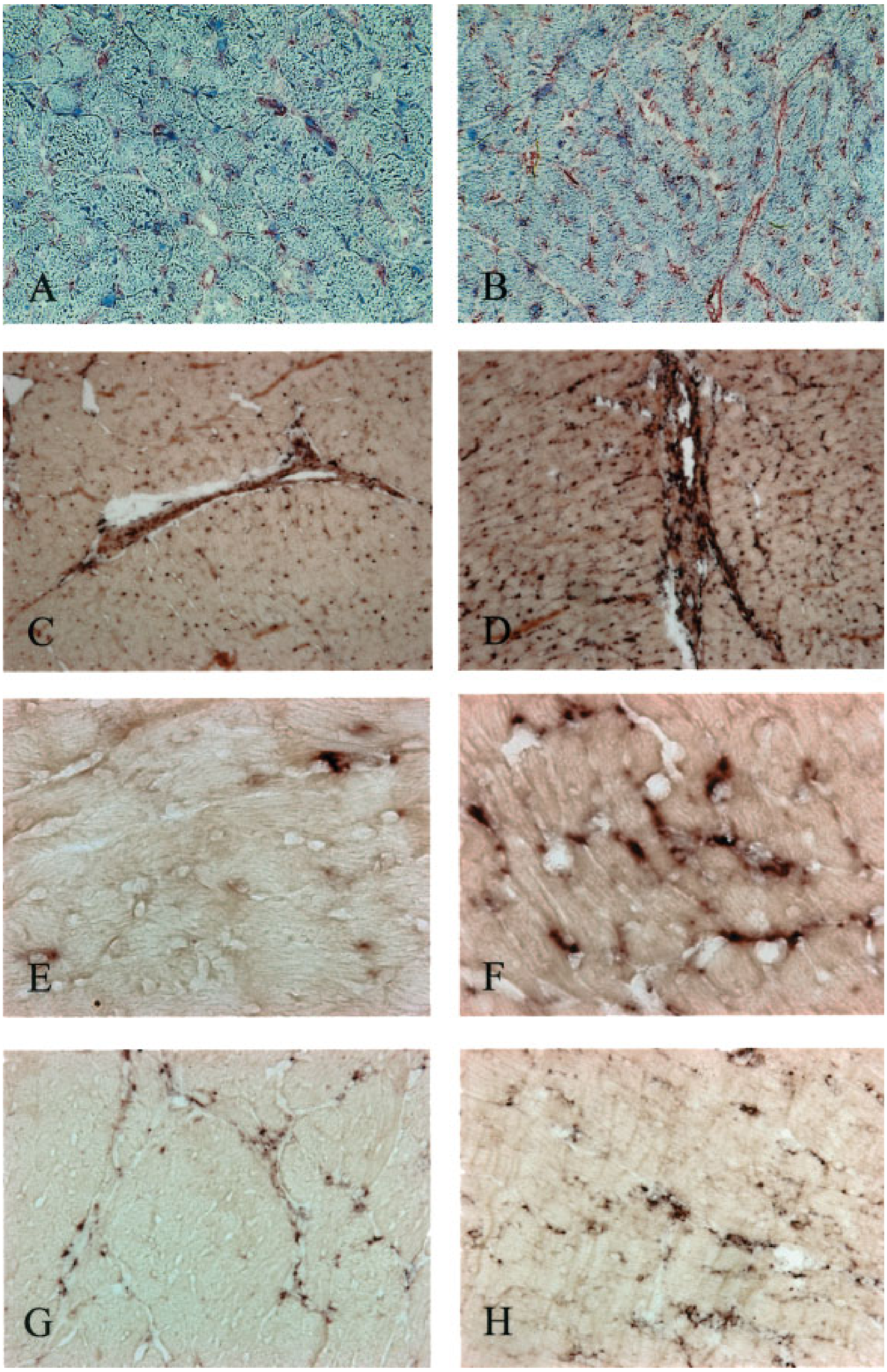

**Figure 3.** Representative immunohistological and *in situ* hybridization data. (A, B) Integrin-$\beta$1 protein expression is higher in the heart of a subtotally nephrectomized rat (B) compared to a sham-operated control animal (A). C–H: *in situ* analysis of angiotensinogen (C, D), *TGF-$\beta$1* (E, F) and endothelin-1 (G, H) mRNA expression by non-radioactive *in situ* hybridization in sham-operated (C, E, G) and subtotally nephrectomized rats (D, F, H). mRNA expression of all three genes is increased in the heart in renal failure

Copyright © 2003 John Wiley & Sons, Ltd. 

*et al.*, 1994). Interestingly, this was not seen in other experimental models of cardiac hypertrophy, i.e. the spontaneously hypertensive rat (SHR) or the 1C-2K rat with renovascular hypertension (Amann *et al.*, 1994; 1998). The pathophysiology of these cardiac alterations is still not clear; however, earlier studies found some evidence for a pathophysiological role of the RAS and the ET system (Amann and Ritz, 1997, 2001; Amann *et al.*, 1998; Wessels *et al.*, 1999).

In the present study, we investigated differential gene expression in the course of cardiac remodelling in experimental renal failure using cDNA array technology. We found an early upregulation of several groups of genes known to be involved in ECM production and remodelling, i.e. collagens, proteoglycans, laminin and connected linker molecules. Some of the results of the present study are in agreement with earlier immunohistochemical and molecular biological studies in the heart of SNX animals of renal failure (Amann *et al.*, 1998; Wessels *et al.*, 1999), or were confirmed by other assays (Figure 3).

### Potential links from the RAS to the extracellular matrix via G-proteins, second messengers and cytoskeleton

In a previous study using *in situ* hybridization we found increased cardiac renin, angiotensinogen and preproendothelin mRNA after induction of renal failure (Amann and Ritz, 1997; Amann *et al.*, 2000). An important role of the RAS in cardiovascular remodelling, in particular cardiac fibrosis, has been documented by experimental and clinical studies (Brilla, 2000). In the present study, we found evidence for an early upregulation of the $ET_B$ receptor in the hearts of SNX after 2 w. The $ET_B$ receptor is predominantly located on endothelial cells and is known to be upregulated in LVH in a G protein-dependent manner (Douglas and Ohlstein, 1997; Zolk *et al.*, 2002).

Therefore, as shown for the kidney, several specific ET responses apparently exist in the heart (Nambi *et al.*, 2001) which represent not only a prohypertrophic stimulus for cardiomyocytes but also stimulate fibroblasts (Tsuruda *et al.*, 2002) and vascular smooth muscle cells (VSMC; Giulumian *et al.*, 2002), as indicated by earlier studies using ET-receptor blockers (Amann *et al.*, 2000; Nabokov *et al.*, 1999).

We found strong evidence for upregulation of several second messenger molecules, i.e. protein kinase C and associated molecules, the phosphatidylinositol pathway, phosphoinositide 3-kinase (PI3K) and phosphoinositide 5-kinase (PI5K), which are known to be connected with actin filament formation (Hartwig *et al.*, 1995; Keely *et al.*, 1997). Interestingly, an increased interstitial expression of integrin-$\beta 1$ after SNX (Figure 2; Amann *et al.*, 1998) confirms its central role in ECM formation and also its mediating function by binding to actin stress fibres.

Since we do not find a remarkable upregulation of actin genes, it remains to be investigated whether actin stress fibre formation can directly be correlated to the increase of second messengers and integrins that we found. We also postulate an important role for phosphorylation and dephosphorylation of phosphatidylinositol in regulation of cardiac remodelling in LVH. This hypothesis will need to be confirmed using more specialized phosphorylation assays.

### Motor proteins play a potential role in signal translation

There is strong experimental evidence that motor proteins are involved in the formation of focal adhesions, integrin assembly and ECM formation, e.g. it has been found that myotonic dystrophy kinase related cdc42-binding kinase (MRCK) phosphorylates myosin light chains (Leung *et al.*, 1998). Myosin light chain phosphorylation promotes both myosin filament assembly and actin-activated myosin ATPase activity (Burridge *et al.*, 1996). These effects result in bundling of actin filaments. The resulting tension is transmitted to integrins, which cluster in a characteristic fashion (Chrzanowska-Wodnicka and Burridge, 1996). As expected, it is mainly the expression of myosin genes and of genes coding for associated proteins, as well as other motor proteins, that are regulated in our experiments. We are currently not able to address any signalling function for dynein and kinesin, but they function as transporters along microtubules and are co-localized with or linked to actin fibres via further linker molecules, respectively (Oakley and Brunette, 1995).

Copyright © 2003 John Wiley & Sons, Ltd.

### Two potential pathways are leading to ECM formation in hearts of renal failure

Whereas most of the collagens and proteoglycans are upregulated during the first days after operation, in the 12 w group a striking upregulation of laminins, together with integrins, is noted. In particular, integrin is thought to be a central player because of its influence on RHO family GTPases. Structural cytoskeletal proteins again are mediators of integrin clustering and microtubule polymerization is affected by *RHO A* (Schoenwaelder and Burridge, 1999). We conclude from our results that at least two pathways for ECM remodelling are operative. They both start with activation of the RAS. Subsequently, either G-proteins and second messengers are activated (short-term signalling) or motor proteins, actins and integrins are upregulated (long-term signalling; Figure 4).

### Many other genes remain to be investigated

Some upregulated ECM genes discussed here were also identified by expression profiling of human hypertrophied cardiac tissue, e.g. myosin regulatory light chains, troponin and desmin (Hwang *et al*., 2000); it should be mentioned, however, that many other molecules appear to be regulated and have to be investigated in further detail. Examples include the TIMP and MMP proteins, which were shown to be important regulators and mediators of renal fibrosis via induction of aldosterone and angiotensin II (ANGII) (Papakonstantinou *et al*., 2001). They were not addressed in the present experiment and have to be investigated in further studies.

Furthermore, members of other gene families are obviously regulated and are potentially involved in cardiac remodelling as shown by us, and others (Hwang *et al*., 2000, 2002). CD59 is an 18–20 kDa GPI-anchored membrane protein that functions as a key regulator of the terminal step of the complement activation cascade. It restricts binding of C9 to the C5b-8 complex, thereby preventing the formation of the membrane attack complex. The rat analogue of human CD59 (protectin) is expressed in the sarcolemmal membranes of normal cardiomyocytes as an important sarcolemmal

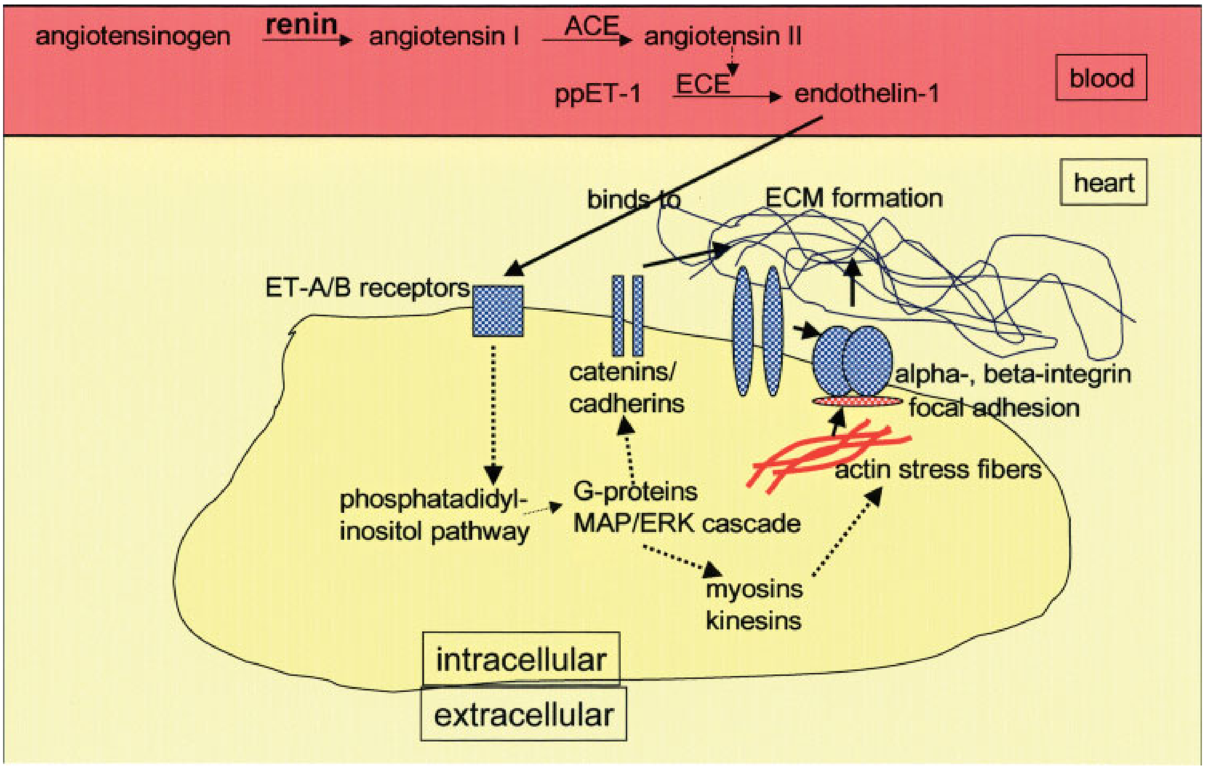

**Figure 4.** Schematic summary of postulated pathways involved in cardiac remodelling of renal failure

Copyright © 2003 John Wiley & Sons, Ltd. 

inhibitor of the complement membrane attack complex (MAC; Vakeva *et al.*, 1994); it is lost, or downregulated, after ischaemic or hypoxic injury to the heart (Venugopal *et al.*, 2001). In addition, in experimental studies it was shown that infusion or overexpression of CD59 offers a significant protection against complement-mediated myocardial injuries (Chakraborti *et al.*, 2000; Fisicaro *et al.*, 2000).

Lipoprotein lipase (LPL) of either VSMC or macrophage origin is an endothelial-bound enzyme that is rate determining for the clearance of triacylglycerol-rich lipoproteins. It is known to accelerate atherosclerosis (Esenabhalu *et al.*, 2002; Wilson *et al.*, 2001), is upregulated in cardiomyocytes by insulin (Ewart *et al.*, 1999) and its heparin-releasable LPL activity was higher in the heart of rats with moderate, but not severe, diabetes (Rodrigues *et al.*, 1997).

Heat shock proteins (*HSP*s) are well known for their ability to 'protect' the structure and function of native macromolecules, particularly as they traffic across membranes. Some of them (*HSP*27, *HSP*72, *HSP*60) have been shown to be upregulated after cardiac ischaemic/hypoxic injury in order to offer cardioprotection against ischaemic injury by induction of antiapoptotic effects via interaction with *bax* and/or *bak* pathways (Kirchhoff *et al.*, 2002; Lin *et al.*, 2001).

Plasminogen activator inhibitor-1 (*PAI*-1) is a major anti-fibrinolytic glycoprotein thought to promote vascular growth and fibrosis during various pathophysiological situations (Venugopal *et al.*, 2001). In particular, it was shown that aldosterone interacts with *ANG* II to increase *PAI*-1 expression *in vitro* and *in vivo*. *PAI*-1, by inhibiting the production of plasmin from plasminogen, tips the balance in favour of ECM accumulation, thereby promoting fibrosis (Brown *et al.*, 2002).

Ubiquitin, a so-called repair-related protein, is upregulated in the heart early after injury (Ishikawa *et al.*, 2000), particularly in post-ischaemic recovery (Sharma *et al.*, 1996). In addition, the ubiquitin/proteasome system represents the cell's major tool for extralysosomal protein degradation, regulating a variety of different processes, such as proliferation, differentiation, cell cycling and apoptosis. Because both negative and positive regulators of proliferation and apoptosis undergo proteasomal degradation in a tightly regulated and temporally controlled fashion, the 26S proteasome can play opposite roles in the regulation of proliferation and apoptosis (Naujokat and Hoffmann, 2002).

Calcyclin, a member of the S100 protein family of Ca-binding proteins, is expressed in human epithelial cells and fibroblasts of several organs, indicating that it is related either to proliferation rate or secretion activity (Kuznicki *et al.*, 1992). Although not much is known about the specific role of calcyclin in the heart, Ca-binding proteins play a critical role in cardiomyocyte function regulating sarcoplasmic reticulum $Ca^{2+}$ handling (McMahon *et al.*, 2002).

We plan to investigate the signalling pathways leading to ECM formation in more detail. Currently, we are performing cDNA sub-arrays on glass slides and nylon membranes, respectively, containing clones which represent about 1000 genes potentially involved in LVH, and about 100 'housekeeping' genes as controls. With this array, which contains only resequenced clones, we are more flexible than with the global array, in that we can reach a higher sensitivity and can repeat the same experiment several times, which should enable us to detect changes even in genes with low expression.

We further intend to investigate samples obtained at other time points in the course of renal failure, to better characterize the time course. Furthermore, the similarities or differences to other models of LVH and the effect of various treatments will be studied. Proteomic assays, especially those looking for protein modifications such as phosphorylations, are under way and should allow us to obtain further insights into the molecular processes involved in cardiac remodelling in renal failure.

### Acknowledgements

The authors are indebted to Silke Wolterink, Diana Lutz, Heike Ziebart and Stefan Söllner for expert technical assistance, and Martin Holst, Peter Neubert, and Wolfgang Huber for their help with data analysis. We further thank the members of the department of Annemarie Poustka (German Cancer Research Center) for their help with the hybridizations. The rat unigene-1 set was amplified and spotted at the RZPD Berlin (Anja Kellermann, Katja Schäfer, Uwe Radelof). This work was supported by the BMBF (BMBF 01 KW 9501) and the BMBF (IZKF; Project B 40).

Copyright © 2003 John Wiley & Sons, Ltd. 

### References

Amann K, Breitbach M, Ritz E, Mall G. 1998. Myocyte/capillary mismatch in the heart of uremic patients. *J Am Soc Nephrol* **9**: 1018–1022.

Amann K, Koch A, Hofstetter J, *et al*. 2001. Glomerulosclerosis and progression: effect of subantihypertensive doses of $\alpha$- and $\beta$-blockers. *Kidney Int* **60**: 1309–1323.

Amann K, Kronenberg G, Gehlen F, *et al*. 1998. Cardiac remodelling in experimental renal failure — an immunohistochemical study. *Nephrol Dial Transplant* **13**: 1958–1966.

Amann K, Münter K, Wessels S, *et al*. 2000. Endothelin A receptor blockade prevents capillary/myocyte mismatch in the heart of uremic animals. *J Am Soc Nephrol* **11**: 1702–1711.

Amann K, Neimeier KA, Schwarz U, *et al*. 1997. Rats with moderate renal failure show capillary deficit in heart, but not skeletal muscle. *Am J Kidney Dis* **30**: 382–388.

Amann K, Ritz E, Wiest G, Klaus G, Mall G. 1994. A role of parathyroid hormone for the activation of cardiac fibroblasts in uremia. *J Am Soc Nephrol* **4**: 1814–1819.

Amann K, Ritz E. 1997. Cardiac disease in chronic uremia: pathophysiology. *Adv Renal Replace Ther* **4**: 212–224.

Amann K, Ritz E. 2001. The heart in renal failure — a specific uremic cardiomyopathy? *J Clin Bas Cardiol* **4**: 109–113.

Boer JM, Huber WK, Sultmann H, *et al*. 2001. Identification and classification of differentially expressed genes in renal cell carcinoma by expression profiling on a global human 31 500-element cDNA array. *Genome Res* **11**: 1861–1870.

Brilla CG. 2000. Renin–angiotensin–aldosterone system and myocardial fibrosis. *Cardiovasc Res* **47**: 1–3.

Brown NJ, Vaughan DE, Fogo AB. 2002. Aldosterone and PAI-1: implications for renal injury. *J Nephrol* **15**: 230–235.

Burridge K, Chrzanowska-Wodnicka M. 1996. Focal adhesions, contractility and signalling. *Ann Rev Cell Dev Biol* **12**: 463–519.

Cattaruzza M, Eberhardt I, Hecker M. 2001. Mechanosensitive transcription factors involved in endothelin B receptor expression. *J Biol Chem* **276**: 36 999–37 003.

Cau J, Faure S, Comps M, Delsert C, Morin N. 2001. A novel p21-activated kinase binds the actin and microtubule networks and induces microtubule stabilization. *J Cell Biol* **155**: 1029–1042.

Chakraborti T, Mandal A, Mandal M, Das S, Chakraborti S. 2000. Complement activation in heart diseases. Role of oxidants. *Cell Signal* **12**: 607–617.

Chomczynski P, Sacchi N. 1987. Single-step method of RNA isolation by acid guanidinium thiocyanate-phenol–chloroform extraction. *Anal Biochem* **162**: 156–159.

Chrzanowska-Wodnicka M, Burridge K. 1996. *RHO*-stimulated contractility drives the formation of stress fibres and focal adhesions. *J Cell Biol* **133**: 1403–1415.

Douglas SA, Ohlstein EH. 1997. Signal transduction mechanisms mediating the vascular actions of endothelin. *J Vasc Res* **34**: 152–164.

Esenabhalu VE, Cerimagic M, Malli R, *et al*. 2002. Tissue-specific expression of human lipoprotein lipase in the vascular system affects vascular reactivity in transgenic mice. *Br J Pharmacol* **135**: 143–154.

Ewart HS, Carroll R, Severson DL. 1999. Lipoprotein lipase activity is stimulated by insulin and dexamethasone in cardiomyocytes from diabetic rats. *Can J Physiol Pharmacol* **77**: 571–578.

Fisicaro N, Aminian A, Hinchliffe SJ, *et al*. 2000. The pig analogue of CD59 protects transgenic mouse hearts from injury by human complement. *Transplantation* **70**: 963–968.

Giulumian AD, Molero MM, Reddy VB, *et al*. 2002. Role of ET-1 receptor binding and [Ca(2+)](i) in contraction of coronary arteries from DOCA-salt hypertensive rats. *Am J Physiol Heart Circ Physiol* **282**: H1944–1949.

Hartwig JH, Bokoch GM, Carpenter CL, *et al*. 1995. Thrombin receptor ligation and activated *RAC* uncap actin filament barbed ends through phophoinositide synthesis in permeabilized human platelets. *Cell* **82**: 643–653.

Hwang DM, Dempsey AA, Lee CY, Liew CC. 2000. Identification of differentially expressed genes in cardiac hypertrophy by analysis of expressed sequence tags. *Genomics* **66**: 1–14.

Hwang JJ, Allen PD, Tseng GC, *et al*. 2002. Microarray gene expression profiles in dilated and hypertrophic cardiomyopathic end-stage heart failure. *Physiol Genom* **10**: 31–44.

Ishikawa Y, Akasaka Y, Ishii T, *et al*. 2000. Sequential changes in localization of repair-related proteins (heat shock protein 70, ubiquitin and vascular endothelial growth factor) in the different stages of myocardial infarction. *Histopathology* **37**: 546–554.

Keely PJ, Westwick JK, Whitehead IP, Der CJ, Parise LV. 1997. Cdc42 and *RAC* 1 induce integrin-mediated cell motility and invasiveness through PI(3)K. *Nature* **390**: 632–636.

Khurana B, Khurana T, Khaire N, Noegel AA. 2002. Functions of LIM proteins in cell polarity and chemotactic motility. *EMBO J* **21**: 5331–5342.

Kirchhoff SR, Gupta S, Knowlton AA. 2002. Cytosolic heat shock protein 60, apoptosis, and myocardial injury. *Circulation* **105**: 2899–2904.

Krendel M, Zenke FT, Bokoch GM. 2002. Nucleotide exchange factor GEF-H1 mediates cross-talk between microtubules and the actin cytoskeleton. *Nature Cell Biol* **4**: 294–301.

Kuznicki J, Kordowska J, Puzianowska M, Wozniewicz BM. 1992. Calcyclin as a marker of human epithelial cells and fibroblasts. *Exp Cell Res* **200**: 425–430.

Leung T, Chen XQ, Tan I, Manser E, Lim L. 1998. Myotonic dystrophy kinase-related cdc42-binding kinase acts as a cdc42 effector in promoting cytoskeletal reorganization. *Mol Cell Biol* **18**: 130–140.

Lin KM, Lin B, Lian IY, *et al*. 2001. Combined and individual mitochondrial HSP60 and HSP10 expression in cardiac myocytes protects mitochondrial function and prevents apoptotic cell deaths induced by simulated ischaemia-reoxygenation. *Circulation* **103**: 1787–1792.

Mall G, Huther W, Schneider J, Lundin P, Ritz E. 1990. Diffuse intermyocardiocytic fibrosis in uraemic patients. *Nephrol Dial Transpl* **5**: 39–44.

McMahon AC, Greenwald SE, Dodd SM, Hurst MJ, Raine AE. 2002. Prolonged calcium transients and myocardial remodelling in early experimental uraemia. *Nephrol Dial Transplant* **17**: 759–764.

Nabokov AV, Amann K, Wessels S, *et al*. 1999. Endothelin receptor antagonists influence cardiovascular morphology in uremic rats. *Kidney Int* **55**: 512–519.

Nambi P, Clozel M, Feuerstein G. 2001. Endothelin and heart failure. *Heart Fail Rev* **6**: 335–340.

Naujokat C, Hoffmann S. 2002. Role and function of the 26S proteasome in proliferation and apoptosis. *Lab Invest* **82**: 965–980.

Copyright © 2003 John Wiley & Sons, Ltd. 

Oakley C, Brunette DM. 1995. Topographic compensation: guidance and directed locomotion of fibroblasts on grooved micromachined substrata in the absence of microtubules. *Cell Motil Cytoskel* **31**: 45–58.

Papakonstantinou E, Roth M, Kokkas B, Papadopoulos C, Karakiulakis G. 2001. Losartan inhibits the angiotensin II-induced modifications on fibrinolysis and matrix deposition by primary human vascular smooth muscle cells. *J Cardiovasc Pharmacol* **38**: 715–728.

Parfrey PS, Foley RN, Harnett JD, *et al*. 1996. Outcome and risk factors for left ventricular disorders in chronic uraemia. *Nephrol Dial Transpl* **11**: 1277–1285.

Raine AE, Margreiter R, Brunner FP, *et al*. 1992. Report on management in renal failure in Europe, XXII, 1992. *Nephrol Dial Transpl* **7**(suppl. 2): 7–35.

Rodrigues B, Cam MC, Jian K, *et al*. 1997. Differential effects of streptozotocin-induced diabetes on cardiac lipoprotein lipase activity. *Diabetes* **46**: 1346–1353.

Ruilope LM, van Veldhuisen DJ, Ritz E, Lüscher TF. 2001. Renal function: the Cinderella of cardiovascular risk profile. *J Am Coll Cardiol* **38**: 1782–1787.

Schoenwaelder SM, Burridge K. 1999. Bidirectional signalling between the cytoskeleton and integrins. *Curr Opin Cell Biol* **11**: 274–286.

Sharma HS, Stahl J, Weisensee D, Low-Friedrich I. 1996. Cytoprotective mechanisms in cultured cardiomyocytes. *Mol Cell Biochem* **160–161**: 217–224.

Stefanski A, Schmidt KG, Waldherr R, Ritz E. 1996. Early increase in blood pressure and diastolic left ventricular malfunction in patients with glomerulonephritis. *Kidney Int* **50**: 1321–1326.

Tsuruda T, Jougasaki M, Boerrigter G, *et al*. 2002. Cardiotrophin-1 stimulation of cardiac fibroblast growth: roles for glycoprotein 130/leukemia inhibitory factor receptor and the endothelin type A receptor. *Circ Res* **90**: 128–134.

US Renal Data System. 1995. *Causes of Death*. Annual Data Report **14**, The National Institutes of Health, National Institute of Diabetes, Digestive and Kidney Disease: Bethesda, MD; 79–90.

Vakeva A, Laurila P, Meri S. 1992. Loss of expression of protectin (CD59) is associated with complement membrane attack complex deposition in myocardial infarction. *Lab Invest* **67**: 608–616.

Vakeva A, Morgan BP, Tikkanen I, *et al*. 1994. Time course of complement activation and inhibitor expression after ischaemic injury of rat myocardium. *Am J Pathol* **144**: 1357–1368.

Venugopal B, Sharon R, Abramovitz R, Khasin A, Miskin R. 2001. Plasminogen activator inhibitor-1 in cardiovascular cells: rapid induction after injecting mice with kainate or adrenergic agents. *Cardiovasc Res* **49**: 476–483.

Wessels S, Amann K, Törnig J, Ritz E. 1999. Cardiovascular structural changes in uremia — implications for cardiovascular function. *Sem Dialysis* **5**: 1–5.

Wilson K, Fry GL, Chappell DA, Sigmund CD, Medh JD. 2001. Macrophage-specific expression of human lipoprotein lipase accelerates atherosclerosis in transgenic apolipoprotein e knockout mice but not in C57BL/6 mice. *Arterioscler Thromb Vasc Biol* **21**: 1809–1815.

Zolk O, Quattek J, Seeland U, *et al*. 2002. Activation of the cardiac endothelin system in left ventricular hypertrophy before onset of heart failure in TG(mREN2)27 rats. *Cardiovasc Res* **53**: 363–371.

Copyright © 2003 John Wiley & Sons, Ltd. 
